# Association of Circulating Proteins with Death or Lung Transplant in Patients with Idiopathic Pulmonary Fibrosis in the IPF-PRO Registry Cohort

**DOI:** 10.1007/s00408-021-00505-y

**Published:** 2022-01-23

**Authors:** Jamie L. Todd, Megan L. Neely, Robert Overton, Hillary Mulder, Jesse Roman, Joseph A. Lasky, Joao A. de Andrade, Mridu Gulati, Howard Huang, Thomas B. Leonard, Christian Hesslinger, Imre Noth, John A. Belperio, Kevin R. Flaherty, Scott M. Palmer, the IPF-PRO Registry investigators

**Affiliations:** 1 Duke Clinical Research Institute, DUMC Box 103002, Durham, NC 27710 USA; 2 Duke University Medical Center, Durham, NC USA; 3 Jane and Leonard Korman Respiratory Institute, Philadelphia, PA USA; 4 School of Medicine, Tulane University, New Orleans, LA USA; 5 Vanderbilt University School of Medicine, Nashville, TN USA; 6 Yale School of Medicine, New Haven, CT USA; 7 Houston Methodist Hospital, Houston, TX USA; 8 Boehringer Ingelheim Pharmaceuticals, Inc, Ridgefield, CT USA; 9 Boehringer Ingelheim Pharma GmbH & Co. KG, Biberach, Germany; 10 Division of Pulmonary and Critical Care Medicine, University of Virginia, Charlottesville, VA USA; 11 David Geffen School of Medicine at UCLA, Los Angeles, CA USA; 12 Division of Pulmonary and Critical Care Medicine, University of Michigan, Ann Arbor, MI USA

**Keywords:** Biomarkers, Interstitial lung diseases, Observational study, Proteomics

## Abstract

Idiopathic pulmonary fibrosis (IPF) is a progressive and ultimately fatal disease with a variable clinical course. Biomarkers that predict patient outcomes are needed. We leveraged data from 300 patients in the multicenter IPF-PRO Registry to determine associations between circulating proteins and the composite outcome of respiratory death or lung transplant. Plasma collected at enrollment was analyzed using aptamer-based proteomics (1305 proteins). Over a median follow-up of 30.4 months, there were 76 respiratory deaths and 26 lung transplants. In unadjusted univariable analyses, 61 proteins were significantly associated with the outcome (hazard ratio > 2 or < 0.5, corrected *p* ≤ 0.05). In multivariable analyses, a set of 4 clinical measures and 47 unique proteins predicted the probability of respiratory death or lung transplant with an optimism-corrected C-index of 0.76. Our results suggest that select circulating proteins strongly associate with the risk of mortality in patients with IPF and confer information independent of clinical measures.

## Introduction

Idiopathic pulmonary fibrosis (IPF) is a progressive disease with a variable clinical course but poor prognosis [1]. Several clinical and radiological characteristics have been associated with mortality in patients with IPF; however, the course of disease for an individual patient remains difficult to predict [2]. The identification and validation of blood biomarkers that are predictive of clinically relevant outcomes in patients with IPF would be of value in improving patient care.

Proteomic profiling plays an important role in the discovery of biomarkers. Patients with IPF have been shown to have a unique peripheral blood proteome [3, 4]. Furthermore, a recent report suggested that the circulating proteome may differentiate patients with IPF who will experience progression over the following 80 weeks from those who will remain stable over this period [5]. We examined the associations between circulating proteins and respiratory death or lung transplant, and the variable importance of circulating proteins as predictors of this outcome, in a cohort of patients from the IPF-PRO Registry.

## Methods

### Study Cohort

The IPF-PRO Registry is a multi-center US registry that enrolled patients with IPF that was diagnosed or confirmed at the enrolling center in the past 6 months, based on the 2011 ATS/ERS/JRS/ALAT diagnostic guidelines [6]. The design of the IPF-PRO Registry has been described [7]. The current analyses were based on data from 300 patients enrolled between March 2016 and February 2017. Outcomes were ascertained from enrollment to June 2019.

### Proteomic Assays

Plasma samples taken at enrollment were assayed using an aptamer-based platform encompassing 1305 proteins (SOMAscan, SOMALogic Inc., Boulder, CO). Protein data were log_2_ transformed prior to analysis.

### Analyses

The univariable association between each protein and the composite outcome of respiratory death or lung transplant was determined using Cox proportional hazards modelling. Linearity and proportional hazards assumptions were assessed prior to fitting each model. Analyses were performed in an unadjusted fashion and adjusted for sex, age, % predicted forced vital capacity (FVC), % predicted diffusing capacity for carbon monoxide (DLco), oxygen use at rest and oxygen use with activity (all assessed at enrollment). *p*-values were corrected for multiple comparisons using the Benjamini–Hochberg method to control the false discovery rate (FDR) at 5%. Proteins for which the hazard ratio was > 2 or < 0.5 and the FDR-corrected p-value was ≤ 0.05 were regarded as significantly associated with the outcome.

Multivariable analyses using Cox regression modelling with the elastic net penalty identified a set of candidate predictors for the composite outcome of respiratory death or lung transplant. First, only proteins were considered in the pool of potential predictors and second, both proteins and clinical factors (sex, age, % predicted FVC, % predicted DLco, oxygen use at rest, oxygen use with activity [all assessed at enrollment]) were considered. The variable importance of the predictors selected by each model was plotted. The performance of each model was assessed using Harrell’s C-index and the optimism-corrected C-index. For the model including both proteins and clinical factors, the C-indices were also computed in groups based on antifibrotic drug use (*i.e*. taking or not taking an approved antifibrotic drug for IPF at enrollment). A multivariable model that included only the clinical factors was constructed and the C-index computed, such that its performance could be compared with that of the protein-inclusive models.

## Results

### Cohort

A total of 300 patients were included. At enrollment, median (Q1, Q3) age was 70 (65, 75) years, 74% were male, 94% were white, 99% were former or current smokers. Median (Q1, Q3) FVC % predicted and DLco % predicted were 69.7 (61.0, 80.2) and 40.5 (31.1, 49.3), respectively. The majority of patients were taking an approved antifibrotic medication for IPF (35% pirfenidone, 19% nintedanib). Median (Q1, Q3) duration of follow-up was 30.4 (20.1, 41.1) months. In total, 76 respiratory deaths and 26 lung transplants occurred.

### Relationship Between Circulating Proteins and Respiratory Death or Lung Transplant

In unadjusted univariable analyses, 61 proteins were significantly associated with the composite of respiratory death or lung transplant. After adjustment for clinical factors, 22 proteins remained significantly associated with the composite outcome (Table 1).

**Table 1:** Circulating proteins associated with respiratory death or lung transplant in patients with IPF in univariable analyses adjusted for clinical factors

Protein	Functions^b^	Risk of death or lung transplant	Adjusted HR^c^ (95% CI)	FDR-corrected *p*-value
C10orf54	Immunoregulatory receptor that inhibits T-cell response. May stimulate MMP14-mediated MMP2 activation	Increased	2.10 (1.34, 3.29)	0.049
CRLF2	Forms a functional complex with TSLP and IL7R that is capable of stimulating cell proliferation through activation of STAT3 and STAT5. Also activates JAK2		2.02 (1.39, 2.94)	0.026
AHSG	Promotes endocytosis, possesses opsonic properties and influences the mineral phase of bone	Decreased	0.17 (0.06, 0.44)	0.026
TYRO3	Receptor tyrosine kinase that transduces signals from the extracellular matrix into the cytoplasm. Regulates physiological processes including cell survival, migration and differentiation		0.33 (0.18, 0.63)	0.040
ADAMTS13	Cleaves von Willebrand factor multimers in plasma, controlling platelet thrombus formation		0.42 (0.27, 0.65)	0.024
RGMA	Bone morphogenetic protein co-receptor that may signal through SMAD1, SMAD5 and SMAD8		0.23 (0.10, 0.53)	0.040
CTSZ	Exhibits carboxy-monopeptidase as well as carboxy-dipeptidase activity. Capable of producing kinin-potentiating peptides		0.47 (0.30, 0.74)	0.050
DPT	Seems to mediate adhesion by cell surface integrin binding. May serve as a communication link between the dermal fibroblast and extracellular matrix. Enhances TGFB1 activity. Inhibits cell proliferation. Accelerates collagen fibril formation and stabilizes collagen fibrils		0.42 (0.26, 0.67)	0.026
CNTF	Survival factor for various neuronal cell types		0.27 (0.13, 0.59)	0.049
ERBB3	Tyrosine-protein kinase that acts as cell surface receptor for neuregulins		0.25 (0.12, 0.53)	0.026
CDH5	Cadherins are calcium-dependent cell adhesion proteins that preferentially interact with themselves in a homophilic manner in connecting cells		0.37 (0.20, 0.68)	0.050
CKM^a^	Reversibly catalyzes transfer of phosphate between ATP and phosphogens (e.g. creatine phosphate). Creatine kinase isoenzymes play a central role in energy transduction	Association varies by protein level (low vs high)	< 8.9: 0.27 (0.10, 0.72)	0.049
			8.9–9.5: 3.22 (0.99, 10.4)	
			> 9.5: 0.13 (0.04, 0.43)	
PAK6^a^	Serine/threonine protein kinase that plays a role in the regulation of gene transcription		< 12.5: 1.25 (0.86, 1.82)	0.024
			12.5–14.6: 0.72 (0.50, 1.06)	
			> 14.6: 3.49 (1.92, 6.33)	
IFNGR2^a^	Associates with IFNGR1 to form a receptor for the cytokine interferon gamma. Ligand binding stimulates activation of the JAK/STAT signaling pathway		< 9.2: 0.17 (0.06, 0.51)	0.026
			> 9.2: 1.87 (1.37, 2.55)	
FASLG^a^	Cytokine that binds to TNFRSF6/FAS, a receptor that transduces the apoptotic signal into cells		< 9.1: 0.31 (0.16, 0.61)	0.050
			> 9.1: 1.64 (1.26, 2.13)	
CD48^a^	Ligand for CD2. Might facilitate interaction between activated lymphocytes. Probably involved in regulating T-cell activation		< 9.3: 0.02 (0.00, 0.14)	0.024
			> 9.3: 1.43 (0.52, 3.88)	
CSF3^a^	Granulocyte/macrophage colony-stimulating factors (CSFs) are cytokines that control the production, differentiation, and function of granulocytes and monocytes-macrophages. This CSF induces granulocytes		< 10.5: 0.50 (0.35, 0.73)	0.024
			> 10.5: 2.01 (1.30, 3.10)	
KLK7^a^	May catalyze the degradation of intercellular cohesive structures in the skin	Association varies over follow-up time	At 12 months: 0.93 (0.61, 1.42)	0.026
			At 24 months: 1.85 (1.08, 3.16)	
			At 36 months: 2.76 (1.41, 5.39)	
RGMB^a^	Member of the repulsive guidance molecule (RGM) family that contributes to the patterning of the developing nervous system		At 12 months: 0.45 (0.24, 0.86)	0.049
			At 24 months: 0.96 (0.44, 2.11)	
			At 36 months: 1.48 (0.55, 4.00)	
TGFBR3^a^	Binds to TGF-β. Could be involved in capturing and retaining TGF-β for presentation to signaling receptors		At 12 months: 0.45 (0.24, 0.85)	0.049
			At 24 months: 0.76 (0.32, 1.80)	
			At 36 months: 1.04 (0.36, 3.00)	
EPHB6^a^	Kinase-defective receptor for members of the ephrin-B family. Modulates cell adhesion and migration. Inhibits JNK activation, T-cell receptor-induced IL-2 secretion and CD25 expression upon stimulation with ephrin-B2		At 12 months: 0.33 (0.16, 0.70)	0.049
			At 24 months: 0.97 (0.44, 2.15)	
			At 36 months: 1.80 (0.64, 5.06)	
DSC2^a^	Component of intercellular desmosome junctions. Involved in the interaction of plaque proteins and intermediate filaments mediating cell–cell adhesion		At 12 months: 0.49 (0.31, 0.78)	0.033
			At 24 months: 0.70 (0.39, 1.27)	
			At 36 months: 0.87 (0.42, 1.80)	

^a^Analyte failed linearity or proportional hazards assumption. For analytes that failed the linearity assumption, the hazard ratio associated with the maximum relative effect from 2–3 piece-wise linear (PWL) components used to represent this analyte is shown. For analytes that failed the proportional hazards assumption, the time-dependent hazard ratio associated with the maximum relative effect at 12, 24, or 36 months is shown. For analytes that failed both, the maximum hazard ratio associated with PWL components at 12, 24, or 36 months is shown

^b^Based on UniProt (https://www.uniprot.org/; accessed February 2021)

^c^Adjustment variables included sex, age, % predicted FVC, % predicted DLco, oxygen use, all assessed at enrolment

**Fig. 1:**
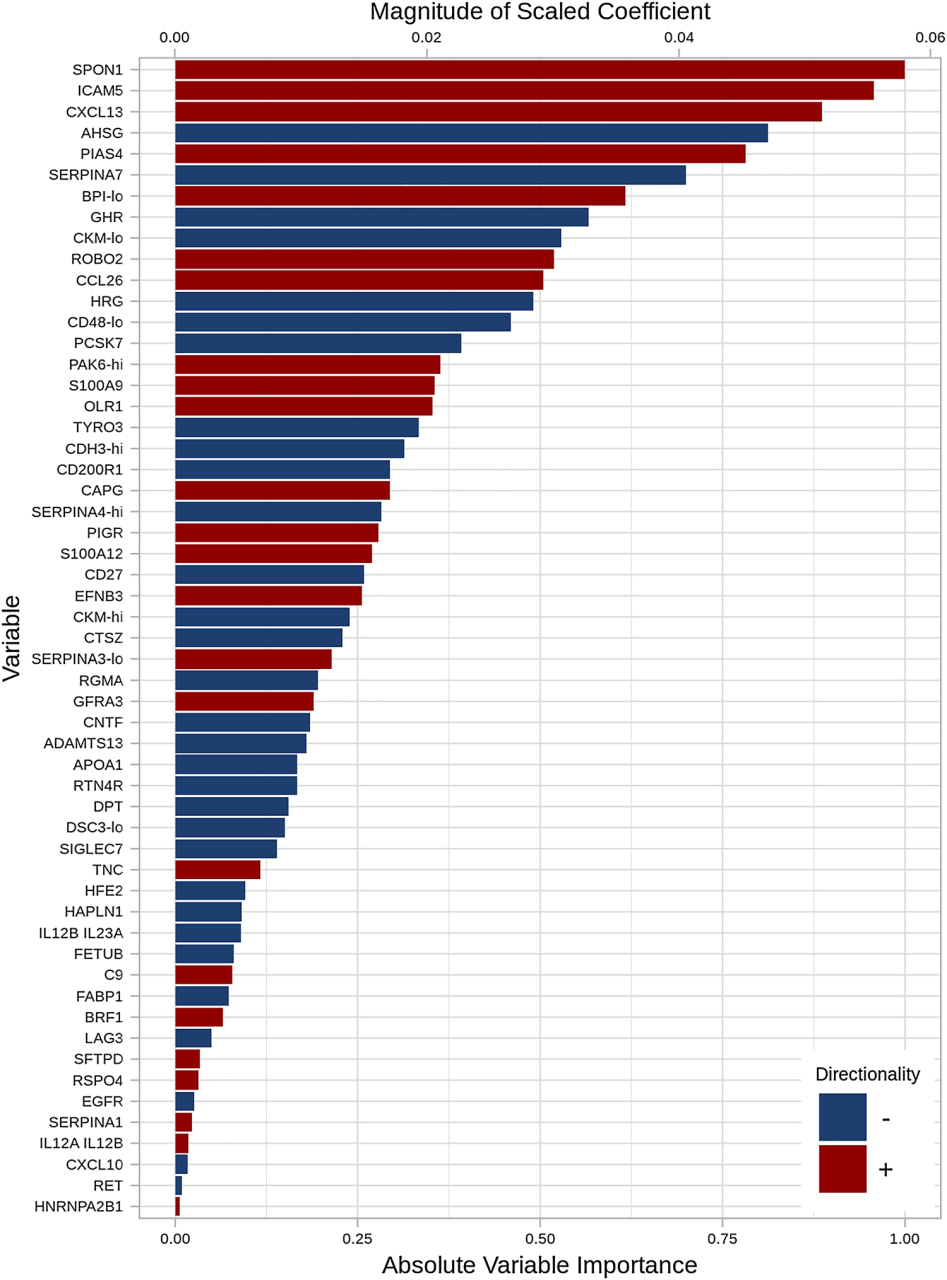
Variable importance of 54 proteins selected using a multivariable model to identify candidate proteins associated with the outcome of respiratory death or lung transplant in patients with IPF. For CKM, two piece-wise linear components are shown

In multivariable analyses considering proteins only, a set of 54 proteins predicted the probability of the composite of respiratory death or lung transplant with a C-index of 0.83 (optimism-corrected C-index of 0.76). The variable importance of the selected proteins is shown in Fig. 1. Among the proteins of greatest importance in discriminating the outcome were spondin-1 (SPON1), intracellular adhesion molecule 5 (ICAM5), C-X-C motif chemokine 13 (CXCL13), alpha 2 HS glycoprotein (AHSG) and protein inhibitor of activated STAT4 (PIAS4).

**Fig. 2:**
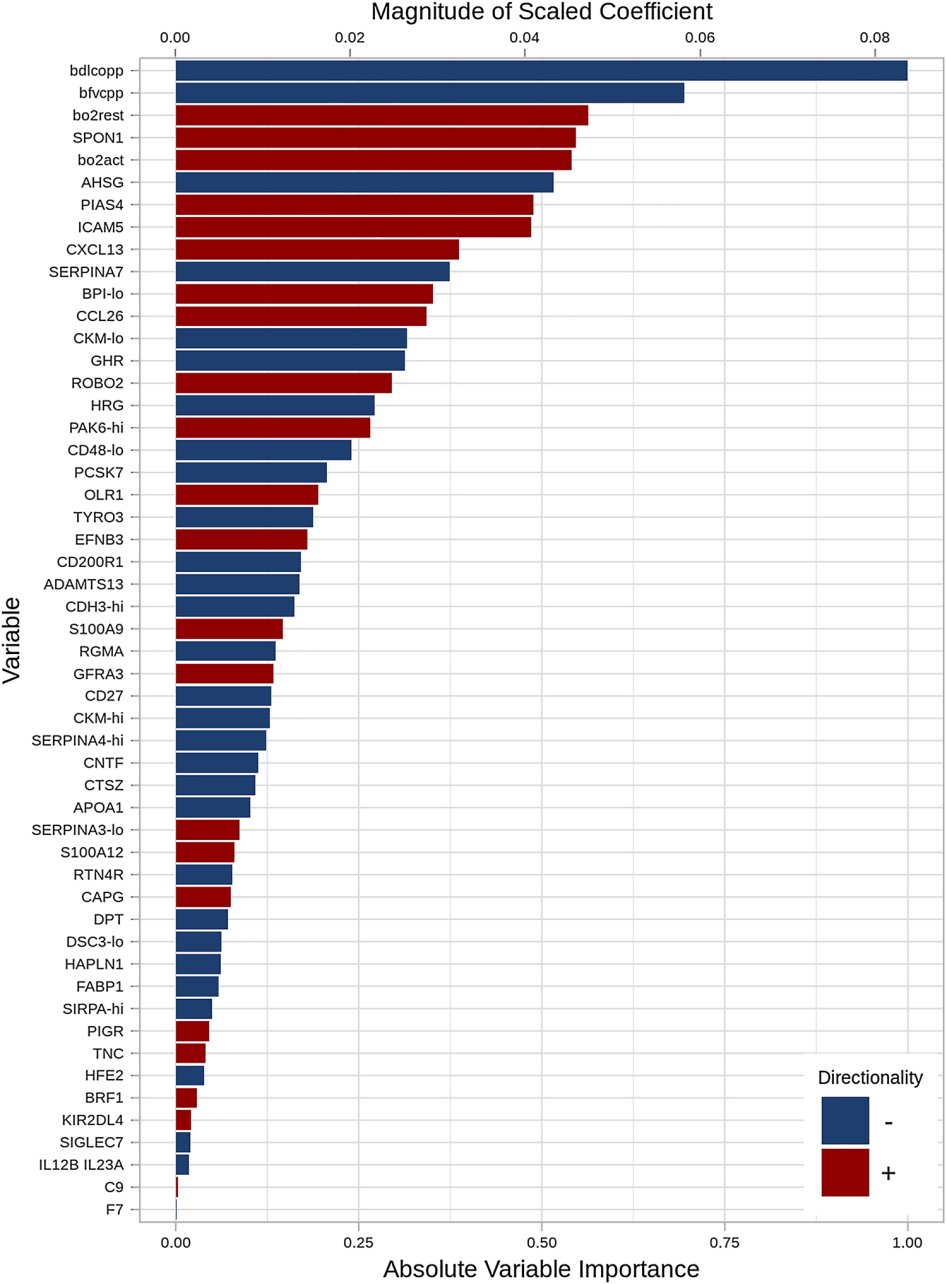
Variable importance of 47 proteins and 4 clinical factors selected using a multivariable model to identify candidate predictors of the outcome of respiratory death or lung transplant in patients with IPF. For CKM, two piece-wise linear components are shown

Multivariable analyses considering both proteins and clinical factors identified a set of 51 predictors (47 proteins, 4 clinical factors) with a C-index of 0.84 (optimism-corrected C-index of 0.76). Model performance was similar in patients who were and were not taking antifibrotic therapy at enrollment (C-index 0.84 and optimism-corrected C-index 0.77 in treated patients; C-index 0.82 and optimism-corrected C-index 0.74 in untreated patients). The variable importance of the selected predictors is shown in Fig. 2. In general, the same protein predictors were retained, but all were of lower importance than oxygen use and measures of lung function. Notably, the performance of the model including both proteins and clinical factors was superior to a model that considered only the clinical factors, for which the C-index was 0.75 and the optimism-corrected C-index was 0.73.

## Discussion

In this analysis of data from 300 patients with IPF, we identified several circulating proteins that strongly associated with a composite outcome of respiratory death or lung transplant, after adjusting for clinical variables known to be associated with mortality in this population [8]. Many of these proteins have functions in inflammation, immune activation/regulation, cell–cell adhesion, or pathways reported to play a role in fibrogenesis (e.g. TGF-β signaling, bone morphogenetic protein signaling, Janus kinase signaling).

While some of our findings are consistent with previous data, such as the association between elevated levels of chemokine CXCL13 and reduced survival [9], our analyses identified several additional candidate proteins as biomarkers of mortality risk, including proteins not measured in previous studies. These results extend previous analyses of data from the IPF-PRO Registry that identified several proteins that associated with clinical measures of IPF severity (% predicted FVC, % predicted DLco, composite physiologic index) at enrollment [3]. In the current analyses, each of the proteins that was associated with all three disease severity measures in this prior work (SPON1, ICAM5, roundabout homolog-2 [ROBO2], polymeric immunoglobulin receptor [PIGR]) was selected by the multivariable model that considered both proteins and clinical factors. While none of these proteins has been well characterized in lung fibrosis, it has been shown that ROBO2 is overexpressed in a mouse model of toxin-induced liver fibrosis, and that the interaction between ROBO2 and its ligand promotes fibrogenic activity within stellate cells [10]. Notably, inclusion of the proteins along with the clinical measures enhanced the discriminatory ability of the model compared with a model that included only clinical factors. This suggests that proteins may confer information that is independent from that captured by measures commonly performed in the clinic.

Among the top protein predictors of the composite of respiratory death or lung transplant were AHSG and PIAS4. Higher AHSG levels and lower PIAS4 levels were associated with reduced risk. These proteins have opposing roles in regulating TGF-β signalling, a pathway known to be important in IPF. Thus it is plausible that they may contribute to the development or progression of IPF. In experimental models, AHSG is an antagonist of TGF-β, with animals genetically lacking in AHSG expression showing increased SMAD2 phosphorylation [11, 12]. Furthermore, TGF-β-mediated suppression of immune cell function was exaggerated in AHSG-deplete animals, as shown by inhibition of macrophage activation [12]. In an experimental model of liver fibrosis, PIAS4 silencing blocked recruitment of SMAD3, decreasing pro-fibrotic gene expression and ameliorating hepatic fibrosis [13]. In the context of these experimental data, our findings compel mechanistic and clinical studies to define the contribution of these proteins to the pathogenesis of IPF and clarify their potential as biomarkers of IPF progression.

Strengths of our analysis include the multi-center nature of the cohort and the adjustment for clinical variables known to influence survival in patients with IPF. Our analyses also have limitations. First, the cohort was a population of mainly white patients enrolled at expert centers in the US, thus our findings may not be applicable to all patients with IPF. Second, while a broad array of proteins were analyzed, some potentially important proteins may have been missed as they were not included on the platform. An aptamer-based approach to protein detection does not always produce results that are reproducible using ELISA and analyses using ELISA are planned.

In conclusion, we identified several novel candidate circulating protein biomarkers for predicting respiratory death or lung transplant in patients with IPF. These data underscore the opportunity to develop biomarker-inclusive algorithms that provide meaningful risk stratification for patients with IPF.

## Data Availability

All the data relevant to the study are included in the article.
