# Correction to: Association of Circulating Proteins with Death or Lung Transplant in Patients with Idiopathic Pulmonary Fibrosis in the IPF-PRO Registry Cohort

**DOI:** 10.1007/s00408-022-00516-3

**Published:** 2022-02-15

**Authors:** Jamie L. Todd, Megan L. Neely, Robert Overton, Hillary Mulder, Jesse Roman, Joseph A. Lasky, Joao A. de Andrade, Mridu Gulati, Howard Huang, Thomas B. Leonard, Christian Hesslinger, Imre Noth, John A. Belperio, Kevin R. Flaherty, Scott M. Palmer, the IPF-PRO Registry investigators

**Affiliations:** 1 Duke Clinical Research Institute, DUMC Box 103002, Durham, NC 27710 USA; 2 Duke University Medical Center, Durham, NC USA; 3 Jane and Leonard Korman Respiratory Institute, Philadelphia, PA USA; 4 School of Medicine, Tulane University, New Orleans, LA USA; 5 Vanderbilt University School of Medicine, Nashville, TN USA; 6 Yale School of Medicine, New Haven, CT USA; 7 Houston Methodist Hospital, Houston, TX USA; 8 Boehringer Ingelheim Pharmaceuticals, Inc, Ridgefield, CT USA; 9 Boehringer Ingelheim Pharma GmbH & Co. KG, Biberach, Germany; 10 Division of Pulmonary and Critical Care Medicine, University of Virginia, Charlottesville, VA USA; 11 David Geffen School of Medicine at UCLA, Los Angeles, CA USA; 12 Division of Pulmonary and Critical Care Medicine, University of Michigan, Ann Arbor, MI USA

## Correction to: Lung 10.1007/s00408-021-00505-y

The article “Association of Circulating Proteins with Death or Lung Transplant in Patients with Idiopathic Pulmonary Fibrosis in the IPF‑PRO Registry Cohort”, written by “Jamie Todd”, was originally published Online First without Open Access. Now the author decided to opt for Open Choice and to make the article an Open Access publication. Therefore, the copyright of the article has been changed to The Author(s) 2022 and the article is forthwith distributed under a Creative Commons Attribution 4.0 International License, which permits use, sharing, adaptation, distribution and reproduction in any medium or format, as long as you give appropriate credit to the original author(s) and the source, provide a link to the Creative Commons licence, and indicate if changes were made. The images or other third party material in this article are included in the article’s Creative Commons licence, unless indicated otherwise in a credit line to the material. If material is not included in the article’s Creative Commons licence and your intended use is not permitted by statutory regulation or exceeds the permitted use, you will need to obtain permission directly from the copyright holder. To view a copy of this licence, visit https://creativecommons.org/licenses/by/4.0.

The original article has been corrected.

